# Multidimensional quantitative characterization of basal cell carcinoma by spectral- and time-resolved two-photon microscopy

**DOI:** 10.1515/nanoph-2023-0722

**Published:** 2024-01-12

**Authors:** Fangyin Guo, Fangrui Lin, Binglin Shen, Shiqi Wang, Yanping Li, Jiaqing Guo, Yongqiang Chen, Yuqing Liu, Yuan Lu, Rui Hu, Jun He, Changrui Liao, Yiping Wang, Junle Qu, Liwei Liu

**Affiliations:** State Key Laboratory of Radio Frequency Heterogeneous Integration (Shenzhen University), Key Laboratory of Optoelectronic Devices and Systems of Guangdong Province and Ministry of Education, College of Physics and Optoelectronic Engineering, Shenzhen University, Shenzhen 518060, China; The Sixth Affiliated Hospital of Shenzhen University and Huazhong University of Science and Technology Union Shenzhen Hospital, Shenzhen, China

**Keywords:** tumor diagnosis, non-invasive, label-free examination, optical imaging, fluorescence lifetime, spectral phasor

## Abstract

Basal cell carcinoma (BCC) is a common type of skin cancer. Conventional approaches to BCC diagnosis often involve invasive histological examinations that can distort or even destroy information derived from the biomolecules in the sample. Therefore, a non-invasive, label-free examination method for the clinical diagnosis of BCC represents a critical advance. This study combined spectral- and time-resolved two-photon microscopy with a spectral phasor to extract rich biochemical information describing macroscopic tumor morphology and microscopic tumor metabolism. The proposed optical imaging technique achieved the rapid and efficient separation of tumor structures in systematic research conducted on normal and BCC human skin tissues. The results demonstrate that a combination of multidimensional data (e.g., fluorescence intensity, spectrum, and lifetime) with a spectral phasor can accurately identify tumor boundaries and achieve rapid separation. This label-free, real-time, multidimensional imaging technique serves as a complement to the conventional tumor diagnostic toolbox and demonstrates significant potential for the early diagnosis of BCC and wider applications in intraoperative auxiliary imaging.

## 1 Introduction

Basal cell carcinoma (BCC) is the predominant type of skin cancer that accounts for approximately 60 % of all skin cancer cases and ranks among the most frequently diagnosed types of cancers globally [1], [2], [3]. Thus, an accurate and timely diagnosis of BCC is crucial in clinical medicine. Instances of BCC are primarily observed in light-skinned individuals, particularly those who have experienced prolonged exposure to ultraviolet radiation. These tumors are typically manifested as skin patches that are pearly in appearance and accompanied by capillary dilation; erosion or ulceration may also occur [4]. Histological examination is currently the preferred method for diagnosing skin cancer type and stage using hematoxylin and eosin (H&E)-stained sections to analyze the structural and morphological changes in pathological tissues [5], [6]. However, the accuracy of histological diagnosis is highly dependent on the expertise and clinical experience of the pathologist. Furthermore, histological processing is time-consuming and potentially changes the macrostructure of the analyzed tissue [7], [8]. Critically, several subtypes of BCC have a significant probability of recurrence after surgery, primarily owing to underestimation of the surgical margins particularly in cases where the tumor is located deeply or the margins are poorly delineated [9], [10]. Therefore, a convenient and efficient detection method is urgently needed for the diagnosis of BBC and accurate determination of the associated tumor boundaries.

Various novel optical imaging techniques have been developed for cancer diagnosis, including optical coherence tomography (OCT) [8], [11], second-harmonic generation (SHG) microscopy [12], two-photon excited fluorescence (TPEF) microscopy [13], [14], fluorescence lifetime imaging microscopy (FLIM) [15], and fluorescence spectrum analysis [16]. Based on the principle of optical coherence, OCT enables the acquisition of micrometer-resolution images within a specific depth of living tissue through the interference of weakly coherent light. Although the application of OCT for noninvasive testing in medicine is widespread, its multidimensional datasets require complex algorithms. The SHG method considers the non-centrosymmetric structure of the material to provide a high-signal-to-noise ratio image. Not only does TPEF enable the morphological analysis of various endogenous fluorophores but it also provides superior tissue penetration depth with extremely low photobleaching and photodamage [14], [17]. In addition to these morphological analysis methods, the study of changes in the tumor microenvironment to characterize the different metabolic pathways of the cells can help to distinguish tumors from healthy tissue. This can be accomplished efficiently using FLIM, which is capable of quantitatively measuring parameters including the oxygen content, ion concentration, pH, and refractive index by characterizing the fluorophore microenvironment and measuring the fluorescence decay rate [16], [18], [19]. This technique is insensitive to factors such as excitation power and fluorophore concentration but is sensitive to changes in the microenvironment of the fluorophore. The high degree of sensitivity and specificity provided by FLIM makes it a promising tool for tissue metabolism assessment and cancer diagnosis. Finally, fluorescence spectrum analysis is widely used to extract and analyze the biochemical information of molecules [16] and localizing fluorophores on the pixel scale by collecting their emission spectra [20].

However, spectral imaging is a complex process owing to the broad and overlapping spectra emitted by various fluorescent groups, making signal separation a challenging endeavor [20], [21]. To address this problem, we previously applied a spectral phasor analysis to rapidly separate and denoise spectrally overlapping fluorescence signals. This approach represents the spectrum of each pixel in an image as a point on the phasor plot through a Fourier transform, simplifying the interpretation and interaction of multidimensional spectral data while compressing three-dimensional data into two-dimensional data [20], [22], [23], [24]. The spectral phasor technique can be used to extract and visualize small variations in the spectrum, thereby providing an option for manual region segmentation. More importantly, it enables rapid and reliable structural segmentation without compromising segmentation quality, eliminating the need for prior knowledge of the spectral shapes and characteristics of various structures [22], [25]. Thus, spectral phasor analyses have been successfully combined with nonlinear optical imaging techniques such as stimulated Raman scattering [25], single-photon imaging [22], and two-photon imaging [26]. These integrations have yielded rapid multicolor imaging results for various applications, including organelle segmentation [25], mouse tissue dynamic imaging [26], and zebrafish embryo structure segmentation [22], [26]. This body of research demonstrates that the spectral phasor analysis is a valuable tool for overcoming the challenges posed by spectral overlap in biological imaging.

In this study, we established a spectral- and time-resolved two-photon microscope system to facilitate BCC analysis and diagnosis. This system integrates four optical imaging modes TPEF, SHG, FLIM, and fluorescence spectrum allowing label-free imaging of human skin tissues. This comprehensive approach enables both qualitative and quantitative analyses of macroscopic morphological features and the tumor microenvironment. By detecting the TPEF signals from flavin adenine dinucleotide (FAD) and the SHG signals from collagen, we directly observe the macromorphological and microstructural changes in skin tissues following lesions [14]. The SHG signals from collagen fibers also facilitate the investigation of fiber breakdown processes during skin tumor invasion. Furthermore, by measuring changes in the fluorescence lifetime, we acquire information describing the metabolism and microenvironment of the tumor region [27]. Under this approach, the fluorescence spectrum serves as a characteristic “fingerprint” of the fluorophores, allowing for the quantitative identification of a variety of fluorophores in the tissue. Finally, the spectral phasor method is applied to simplify the analysis of hyperspectral data and effectively address the spectral overlap in biological tissue imaging. In total, this powerful system enables label-free and real-time investigation of the tissue changes that occur during tumor invasion and growth, which can lead to the accurate determination of tumor boundaries, assist in identifying tumor subtypes, and facilitate studies of cancer growth dynamics.

## 2 Materials and methods

### 2.1 Sample preparation

Normal and diseased human skin specimens were collected from five patients at The Sixth People’s Hospital of Shenzhen. All patients provided informed consent and the study was approved by the hospital ethics committee. Excision and histological examination of the tissue samples were performed by a skilled surgeon. The collected specimens comprised four instances of normal skin tissues and 16 instances of BCC tissues. The surgeon promptly froze the excised tissue samples in liquid nitrogen and stored them at a temperature of −80 °C. The samples were subsequently sliced into multiple sections. Two contiguous sections were used for H&E staining and optical imaging. Routine H&E-stained sections were subjected to standard pathological examinations, histological identification, and diagnosis by a professional dermatology oncologist. The other sections were covered with coverslips and multiple areas within each were selected for two-photon imaging using spectral- and time-resolved microscopy.

### 2.2 Optical setups

**Figure 1:**
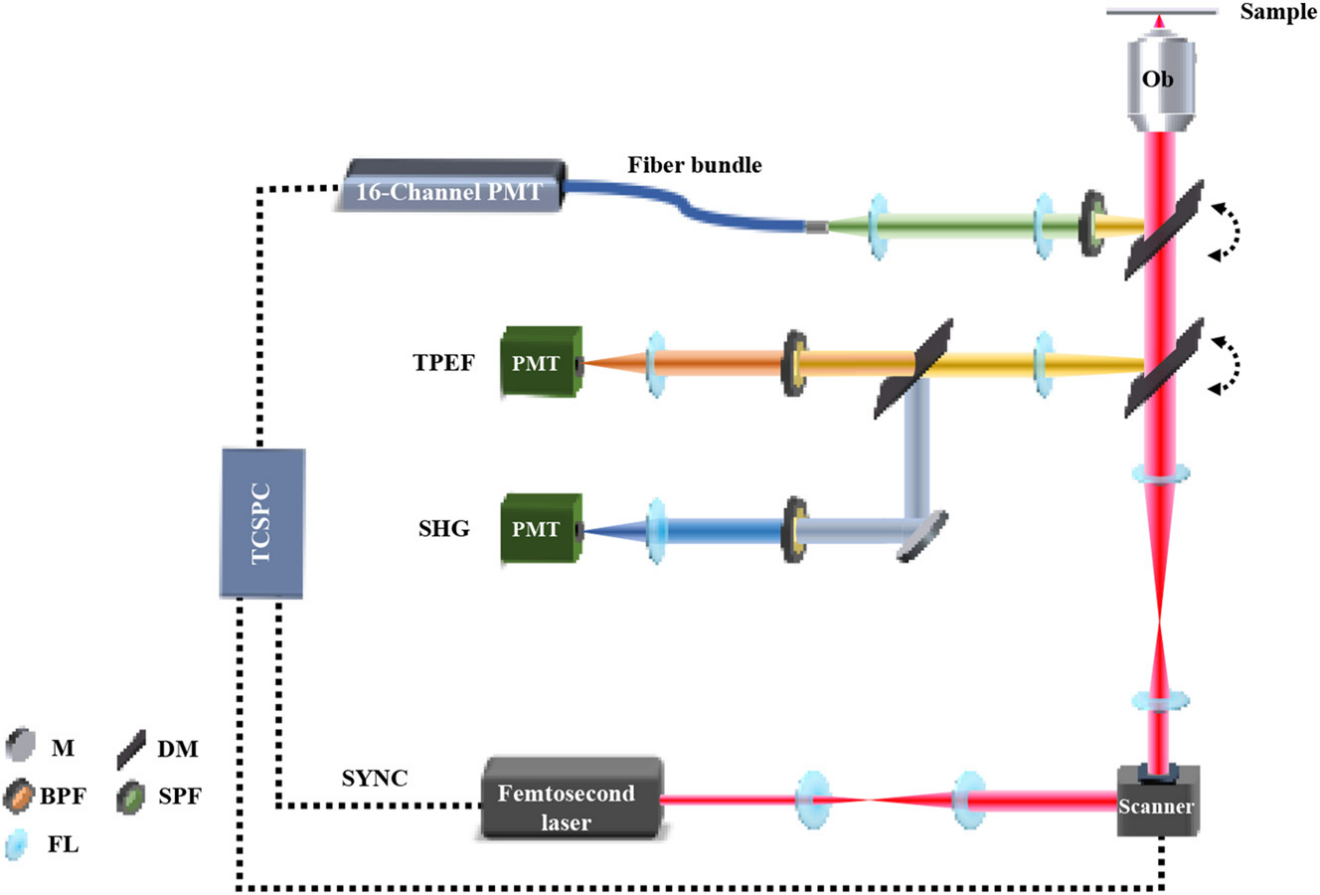
Schematic of spectral- and time-resolved two-photon microscopy system. M, mirror; DM, dichroic mirror; BPF, band-pass filter; SPF, short-pass filter; FL, focal lens.

A 920 nm ultrashort pulse laser with a repetition frequency of 80 MHz (FemtoNL-920nm-1, YSL Photonics) was used as the excitation source. A hybrid (galvano-resonant) scan head (MRD00105, Nikon) was used to achieve high-speed scanning at a maximum speed of 420 fps. A 20× objective lens (CFI Plan Apo *λ* 20×, 0.75NA, Nikon) and a 60× oil immersion objective lens (CFI Plan Apo *λ* 60×, 1.4NA, Nikon) were used to focus the laser on the section and thereby excite the nonlinear signal. The average power at the sample surface was approximately 6 mW. The image size and number of pixels obtained by the 20× objective were 634.88 μm × 634.88 μm and 1024 × 1024 pixels, respectively, 211.63 μm × 211.63 μm and 256 × 256 pixels by the 60× objective. To detect the TPEF, SHG, and spectral signals, we coupled a linear array photomultiplier (PML-16-C, Becker & Hickl GmbH) and two high-sensitivity gallium–arsenide–phosphide photomultipliers (PMT) into a non-descanned light path. Two flip mirrors were used to switch between the detectors. We employed three combinations of LP dichroic mirrors and BP/SP filters to accomplish the different types of imaging: (1) LP 560 and BP 525/50 nm for collecting TPEF signals, (2) LP 495 and BP 460/10 nm for collecting SHG signals, and (3) LP 735 and SP 750 nm for spectrum and lifetime measurements. These signals were fed into the spectrometer through a fiber optic bundle. The spectrometer divided the signals into 16 spectral ranges using a grating with spectral centers ranging from 447.5 to 635 nm in intervals of 12.5 nm. The FAD fluorophore extraction module for lifetime measurement and the time-correlation single-photon counting (TCSPC) module (SPC-150, Becker & Hickl GmbH) were connected to the PMT. Standard samples and probes were used to calibrate the system and thereby ensure the accuracy of the lifetime and spectral measurements while confirming the feasibility of separating probe data using a spectral phasor analysis (Figures S1 and S2 in Supplementary 1) (Figure 1).

### 2.3 Fluorescence spectrum and lifetime measurements

In this study, we investigated the optical properties of BCC using the fluorescence intensity, spectrum, and lifetime. Tissue was categorized as belonging to a non-invasive region (NOIR), near-invasive region (NEIR), or invasive region (IR) based on the following criteria: IR tissues were defined as those with long average fluorescence lifetimes within the tumor boundary; NOIR tissues were defined as those outside the tumor boundary where SHG signals were present; and NEIR tissues were defined as those with a significantly shorter average fluorescence lifetime located between IR and NOIR tissues. To visualize the spectral information and obtain spectrally encoded images, we assigned specific red–green–blue (RGB) color values to each spectral channel. The intensity-weighted color image for each channel was obtained by multiplying the grayscale images acquired from the 16 spectral channels by their corresponding RGB color values. The 16 resulting color images were superimposed to create the final spectrally encoded image. For each of the defined structures (IR, NOIR, and NEIR), we selected the signal intensity of the specified region and integrated it to obtain the corresponding spectral curves. To analyze the variations in these spectra, we divided the SHG signal (Ch 2, 460 ± 6.25 nm) by the maximum TPEF signal channel (Ch 7, 522.5 ± 6.25 nm) to obtain the SHG/TPEF ratio. Additionally, we calculated the TPEF spectral ratio (dividing the signal from spectral channel 4 [Ch 4, 485 ± 6.25 nm] by that from spectral channel 7 [Ch 7, 522.5 ± 6.25 nm]) to analyze the spectral characteristics of different regions [16]. To accomplish these analyses, we developed MATLAB scripts for the selection of corresponding regions, generation of spectral encoding images, and extraction of spectra. To achieve large-field imaging scans, we implemented an autofocus algorithm and incorporated a 20 % overlay that ensured precise and comprehensive image coverage.

The spectral phasor method can significantly enhance the efficiency and intuitiveness of data analysis. This technique employs a Fourier transform to extract the real *G* and imaginary *S* parts of the first harmonic component of the spectrum for each pixel. These two values are represented as coordinates (*G*, *S*) in the phasor plot, effectively integrating the spectral information of each pixel [20]. The transformation equation for the spectral phasor coordinates is as follows [22], [28]:

(1) $$G\left(\lambda \right)=\frac{\overset{\lambda_{\max}}{\underset{\lambda_{\min}}{\sum }}I\left(\lambda \right)\cos\left(\frac{2\pi n\lambda }{\omega }\right)\Delta \lambda }{\overset{\lambda_{\max}}{\underset{\lambda_{\min}}{\sum }}I\left(\lambda \right)\Delta \lambda }$$

(2) $$S\left(\lambda \right)=\frac{\overset{\lambda_{\max}}{\underset{\lambda_{\min}}{\sum }}I\left(\lambda \right)\sin\left(\frac{2\pi n\lambda }{\omega }\right)\Delta \lambda }{\overset{\lambda_{\max}}{\underset{\lambda_{\min}}{\sum }}I\left(\lambda \right)\Delta \lambda }$$

where *λ*_max_ and *λ*_min_ denote the range of the acquired spectra, $I\left(\lambda \right)$ denotes the intensity of the spectral measurement, *n* denotes the number of harmonics, and *ω* is the number of spectral channels. A smaller distance between two points in the phasor plot indicates that they share a higher degree of spectral similarity.

The skin tissue structure was segmented by conducting a spectral phasor analysis on the 16 spectral images and subsequently dividing them into multiple distinct clusters; the literature reports that the number of spectra does not affect the effectiveness of spectral separation [20]. The hyperspectral phase software developed by Cutrale was used to facilitate the analysis of multispectral data obtained from the spectral- and time-resolved imaging systems [22]. Effectively, the use of a spectral phasor in this application facilitates the reduction of multidimensional data (*X*, *Y*, *λ*) to two-dimensional data (*G*, *S*), enabling rapid image segmentation without prior knowledge.

The fluorescence lifetime parameters and fluorescence decay curves were calculated and analyzed for each pixel in the TPEF images, for which the biexponential component fit is expressed as follows [29]:

(3) $$\frac{I\left(t\right)}{I\left(0\right)}=a_{1}\exp\left(-\frac{t}{\tau_{1}}\right)+a_{2}\exp\left(-\frac{t}{\tau_{2}}\right)$$

where *a*_1_, *τ*_1_ and *a*_2_, *τ*_2_ are the amplitude and lifetime values, respectively, of the two different components 1 and 2. The average fluorescence lifetime of each pixel was assigned to a specified RGB color value, and the intensity of this color was weighted to generate a fluorescence lifetime color image, in which the average fluorescence lifetime was calculated as follows [29]:

(4) $$\tau_{\text{mean}}=\frac{a_{1}\tau_{1}+a_{2}\tau_{2}}{a_{1}+a_{2}}$$

The TPEF signal channel with the highest intensity (Ch 7, 522.5 ± 6.25 nm) was chosen for the comprehensive measurement of fluorescence lifetime parameters.

Finally, the SPCImage (Becker& Hickl GmbH) software package was used to analyze the fluorescence decay curves of the FLIM images. Multiple fields of view were selected to image each slice for the collection and statistical analysis of the data. GraphPad Prism was used to perform the statistical analyses of all data in terms of means, analysis of variance (ANOVA), and significant differences.

## 3 Results and discussion

### 3.1 Morphological analysis of skin tissue based on fluorescence intensity

**Figure 2:**
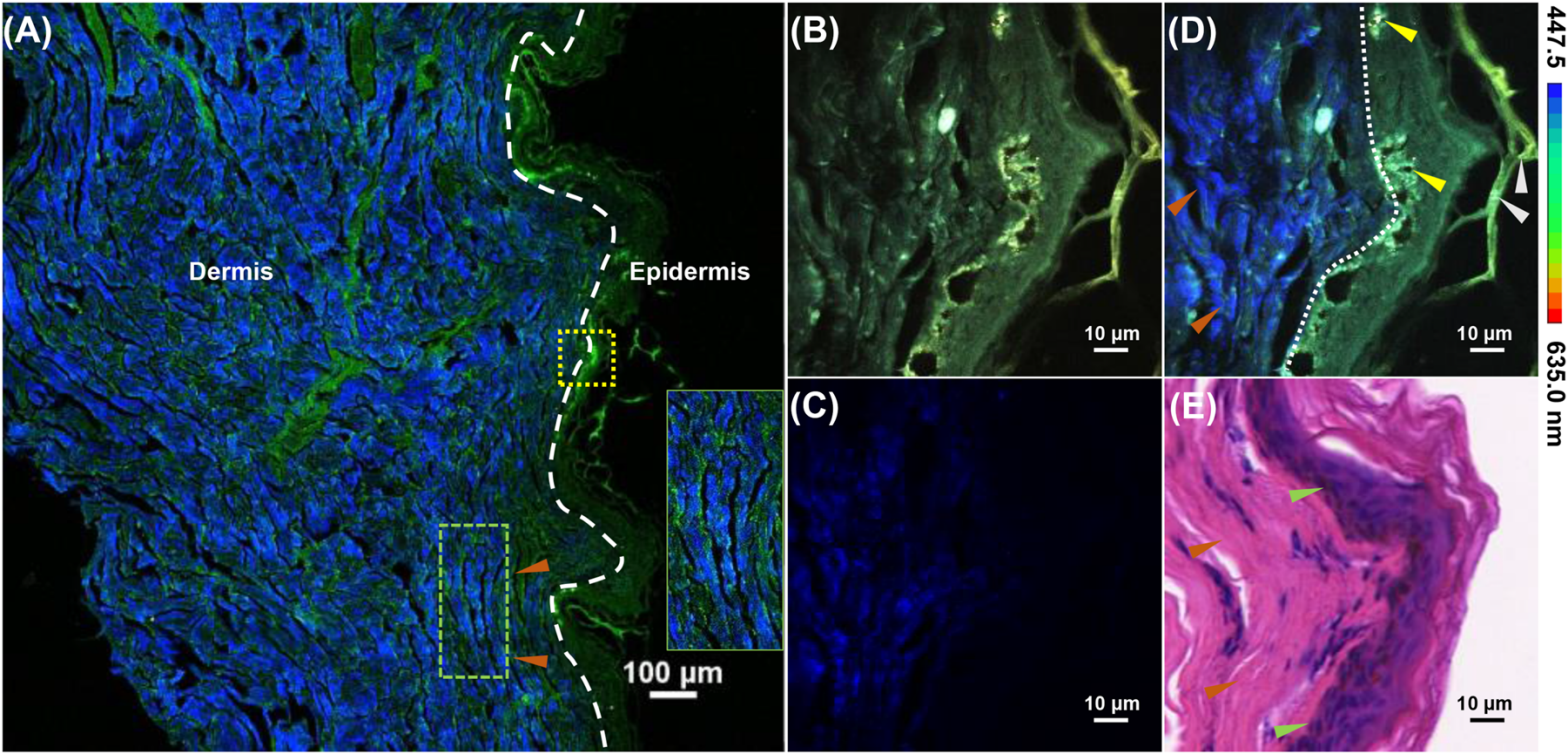
TPEF and SHG imaging of normal human skin tissues and corresponding histological imaging. (A) Large-field image obtained by two-photon and second harmonic scanning imaging; (B) spectrum-coded image of two-photon signals in the yellow box of (A); (C) spectrum-coded image of multichannel superposition; (D) spectrum-coded image of second harmonic signals; (E) histological image of H&E-stained corresponding tissues. In these images, TPEF signals are shown in green, SHG signals are shown in blue, the epidermis and dermis are distinguished by white dotted lines, the green box indicates the location of the enlarged image, the yellow arrow indicates the basal layer, the orange arrow indicates collagen fibers, the gray arrow indicates keratinocytes, and the green arrow indicates melanocytes.

The morphological changes in cancer cells, endogenous fluorophores, and extracellular matrix fibers play crucial roles in cancer research [14], [30]. In this study, we employed spectral- and time-resolved two-photon microscopy to acquire label-free images of normal skin tissues and BCC, thereby enabling the morphological analysis of tumors. The microstructures of the epidermis and dermis of the normal skin tissue are shown in Figure 2. In the large-field scanning image (Figure 2(A)), the strong SHG signal provided distinct contrast, clearly delineating the boundary between the epidermis and dermis, as indicated by the white dashed line. Collagen fibers (orange arrows in Figure 2(A)) are aligned in a parallel pattern (enlarged image in Figure 2(A)), which enables them to provide resilience and strength to the skin. To obtain more detailed information, a smaller region (yellow box in Figure 2(A)) was examined using spectral- and time-resolved microscopy. Figure 2(B) represents the spectral encoding image superimposed on multiple TPEF channels to highlight the specific distribution of FAD. Figure 2(C) shows the collected SHG signals, illustrating the orientations of the collagen fibers in the extracellular matrix (ECM). Finally, we obtained Figure 2(D) by superimposing Figure 2(B) and (C). The melanocytes (yellow arrows in Figure 2(D)) in the epidermis of normal tissues display robust fluorescence intensity. Additionally, significant fluorescence enhancement can be observed in the granules (gray arrows in Figure 2(D)) of the stratum corneum, which are believed to correspond to keratinocytes. Keratinocytes differentiate from human basal stem cells, leading to the expression of structural proteins and culminating in the formation of granular keratinocytes [31], [32]. Histological identification was performed for validation using H&E-stained images of normal tissues (Figure 2(E)). This revealed structurally normal and tightly packed epithelial cells, the presence of melanocytes (green arrows in Figure 2(E)) in the basal lamina, and abundant neatly aligned collagen fibers (orange arrows in Figure 2(E)) in the ECM.

**Figure 3:**
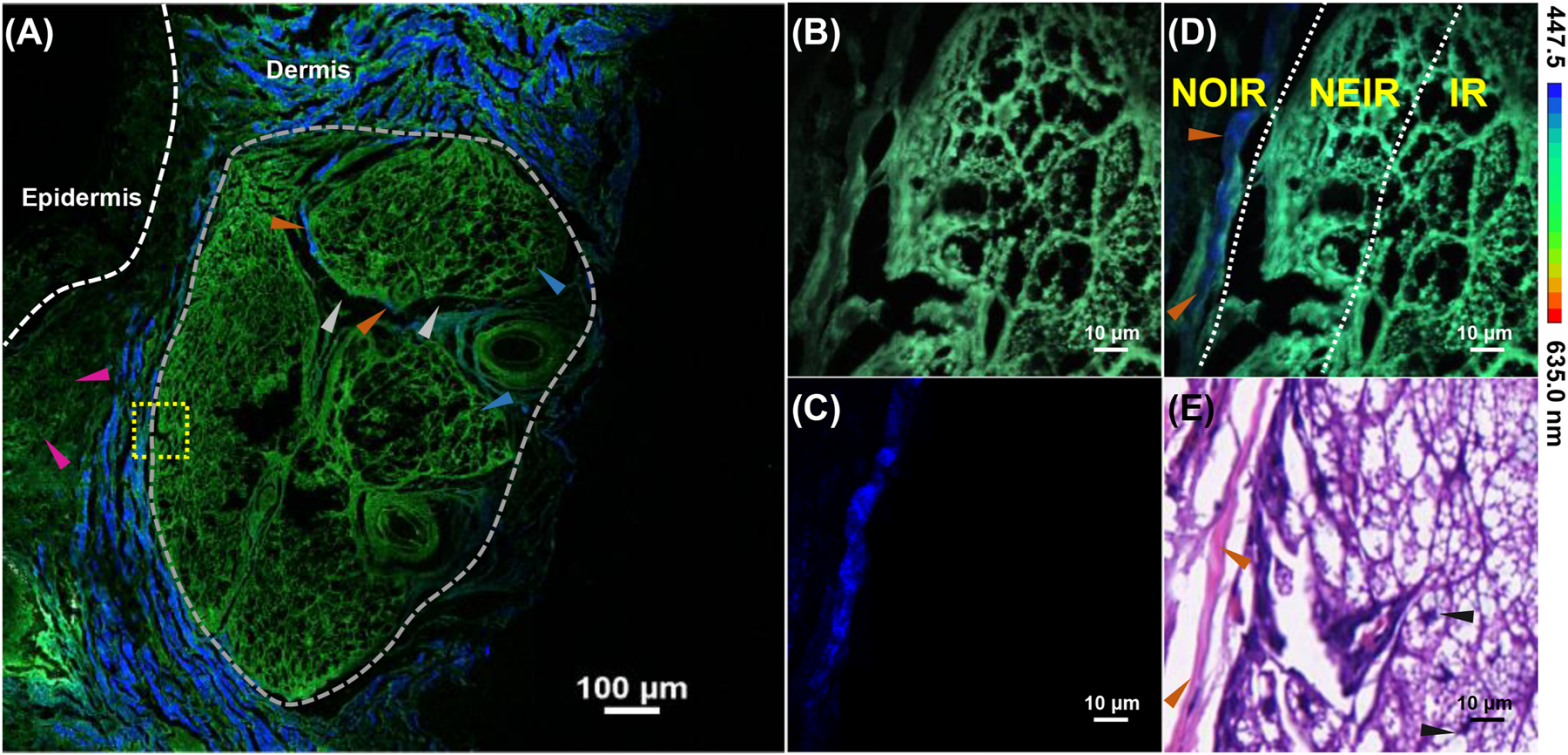
TPEF and SHG imaging of BCC and corresponding histological imaging. (A) Large-field two-photon and second harmonic scanning imaging of the lesion area; (B) spectrum-coded image of two-photon signals in the yellow box in (A); (C) spectrum-coded image of multichannel superimposition; (D) spectrum-coded image of second harmonic signals; (E) histological image of H&E staining. In these images, TPEF signals are shown in green, SHG signals are shown in blue, the epidermis and dermis are distinguished by white dashed lines, the lesion areas are circled by gray dashed lines, the purple arrows indicate areas of fibrolysis, the orange arrows indicate fibers, the blue arrows indicate basal cell lobules, the gray arrows indicate mesenchyme, and the black arrows indicate nuclei.

The distinctive features associated with the structural changes that accompany BCC can be observed by comparing BCC images with those of normal tissue. The optical and corresponding histological images of BCC are presented in Figure 3. As in Figure 2, the white dashed line in Figure 3(A) demarcates the border between the dermis and the epidermis. Notably, cancerous lesions cause heterogeneity in the epidermal layer with irregular epidermal cell contours, enlarged cellular gaps, and invasion into the dermis [14], [33]. Indeed, in Figure 3(A), the SHG signal disappears at certain locations in the dermis (pink arrows), indicating fiber breakdown, and oval nodules filled with mucin and cystic spaces can be observed throughout the dermis (gray dashed lines), consisting of multiple basal cell leaflets (blue arrows). Furthermore, collagen fibers are almost completely broken down, causing the SHG signal to vanish, and only sparsely distributed fibers (yellow arrows in Figure 3(A)) remain between the basal cell lobules connected through the mesenchyme (gray arrows in Figure 3(A)). Figure 3(B)–(D) show the spectrally encoded images, in which the tumor is divided into the NOIR, where collagen fibers are present (orange arrows in Figure 3(D)) and fluorescence lifetimes are long; the IR in the interior of the tumor, where the longest fluorescence lifetimes can be observed; and the NEIR, which is between the two with the shortest fluorescence lifetimes. The white dashed lines Figure 3(D) represent the boundaries between these different regions. Subdividing the tumor into regions may help further explain and investigate the principles underlying changes in the morphologies of tumor regions as well as their surrounding microenvironments. Spectrum-coded images of the tumor regions (Figure 3(D)) reveal the presence of numerous cystic spaces and enhanced TPEF signals stimulated by endogenous FAD, demonstrating the structural diversity of the cancerous region. The spectrum-coded image of BCC is consistent with the corresponding H&E-stained histological image (Figure 3(E)), with visible nuclei in this region (black arrows). Thus, the label-free spectral- and time-resolved imaging model offers significant advantages for the morphological characterization of BCC by eliminating the complex and time-consuming nature of traditional H&E staining, enabling the accurate identification and characterization of skin substructures such as melanocytes, keratinocytes, and collagen fibers.

### 3.2 Microenvironmental analysis of skin tissues based on fluorescence lifetime

When undertaking the pathological diagnosis of a tumor, its cellular metabolism can be studied in addition its morphology [34], [35]. When cells become cancerous, the primary metabolic mode of acquiring adenosine triphosphate (ATP) shifts from oxidative phosphorylation to aerobic glycolysis, resulting in changes in the metabolic state of the cell [36]. To reveal the specific pathological and metabolic features of BCC, we investigated the differences between the redox states of normal and skin tumor cells using the fluorescence lifetime. Specifically, we excited and collected the TPEF signals from FAD and utilized TCSPC to measure the fluorescence lifetime, providing insights into the molecular metabolic states of the cells.

**Figure 4:**
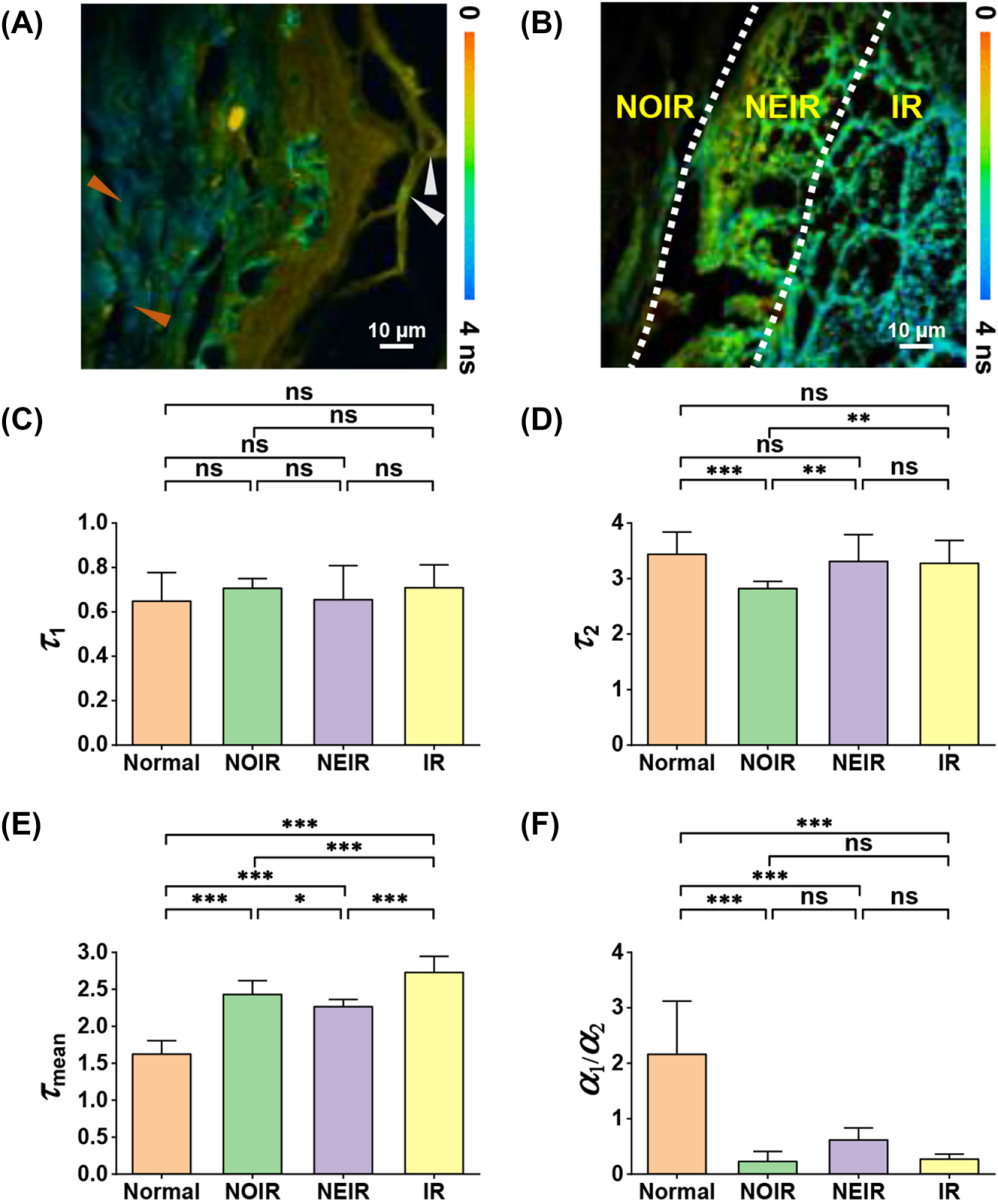
Lifetime-coded images and histograms of normal and diseased human skin tissue. (A) Lifetime-coded image of normal tissue; (B) lifetime-coded image of BCC tissue; (C) fluorescence lifetime of free FAD: *τ*_1_; (D) fluorescence lifetime of protein-bound state FAD: *τ*_2_; (E) average fluorescence lifetime of FAD: *τ*_mean_; (F) ratio of free state FAD to protein-bound state FAD, *a*_1_/*a*_2_. In these images, orange arrows indicate collagen fibers, gray arrows indicate keratinocytes, and the error bars denote the SEM. The notation **** indicates *p* < 0.00001, *** indicates 0.00001 < *p* < 0.0001, ** indicates 0.0001 < *p* < 0.01, * indicates 0.01 < *p* < 0.05, and ns indicates no significant difference via a one-way ANOVA and Tukey’s multiple comparison test.

We selected the Ch 7 signal (522.5 ± 6.25 nm), which exhibited the maximum TPEF signal, to measure the lifetime and calculate the various lifetime parameters for statistical analysis, including the average lifetime of FAD, *τ*_mean_, reflecting the metabolic state and overall changes in the microenvironment; the lifetimes of the two fractions of FAD, characterized by *τ*_1_ and *τ*_2_; and the ratio of free state FAD to protein-bound state FAD, *a*_1_/*a*_2_, allowing the proportions of different metabolic pathways to be investigated [37]. Statistical information describing these four parameters can provides an intuitive understanding of the pathological changes that occur during cancer invasion and growth. Because collagen is broken down after tumor invasion, to maintain the degree of structural similarity, the stratum corneum of normal tissues and BCC were chosen for comparison of their fluorescence lifetime statistics. The results indicate that the average fluorescence lifetimes of the epidermis were significantly different from those of the dermis in normal skin tissues (Figure 4(A)). Notably, areas with SHG signals (orange arrows in Figure 4(A)) overlaying the tissue exhibited prolonged average fluorescence lifetimes. Particles with significantly shorter lifetimes were present in the epidermal stratum corneum (gray arrows in Figure 4(A)), which is consistent with the locations of keratinocytes (gray arrows in Figure 2(C)) in the previous spectral encoding, demonstrating the ability of fluorescence lifetime imaging to discriminate between the microstructures in skin tissue. As shown in Figure 4(B), the fluorescence lifetime within the IR was notably extended compared to that within the normal tissue. This observation suggests a significant alteration in metabolism within the interior of the tumor. Metabolic heterogeneity within a tumor is influenced by multiple factors, including the cellular composition (cancerous and non-cancerous), availability of nutrients, oxygen content, pH, and specific microenvironmental conditions; this can lead to variations in metabolic activity across different regions of the tumor [38].

As shown in Figure 4(C), the *τ*_1_ of the normal tissue exhibited no significant difference compared to the three tumor regions. Similarly, Figure 4(D) shows that no distinctive features were found between the multiple regions for *τ*_2_. However, comparing the *τ*_mean_ of each region (Figure 4(E)), the normal tissue clearly has the shortest fluorescence lifetime, and the differences between the *τ*_mean_ values for three lesion regions are highly significant, with that for the NEIR being significantly lower than that for the NOIR or IR. These differences indicate different metabolic states in the different regions. The *a*_1_/*a*_2_ of the normal tissue (Figure 4(F)) is significantly higher than that of the three tumor regions, indicating that normal tissue is dominated by aerobic metabolism [14]. Additionally, the *a*_1_/*a*_2_ of the NEIR is higher than that of the IR, suggesting that the tumor border has a lower degree of glycolysis than the tumor interior, and a significant portion of metabolism remains in the aerobic form [37]. These differences were found to be statistically significant by the multiple comparison test, demonstrating that fluorescence lifetime can be used to reveal microenvironmental and metabolic differences between normal tissues and diseased regions, distinguish between different structures, and aid in the identification of tumor boundaries.

### 3.3 Quantitative spectral characterization of skin tissue

**Figure 5:**
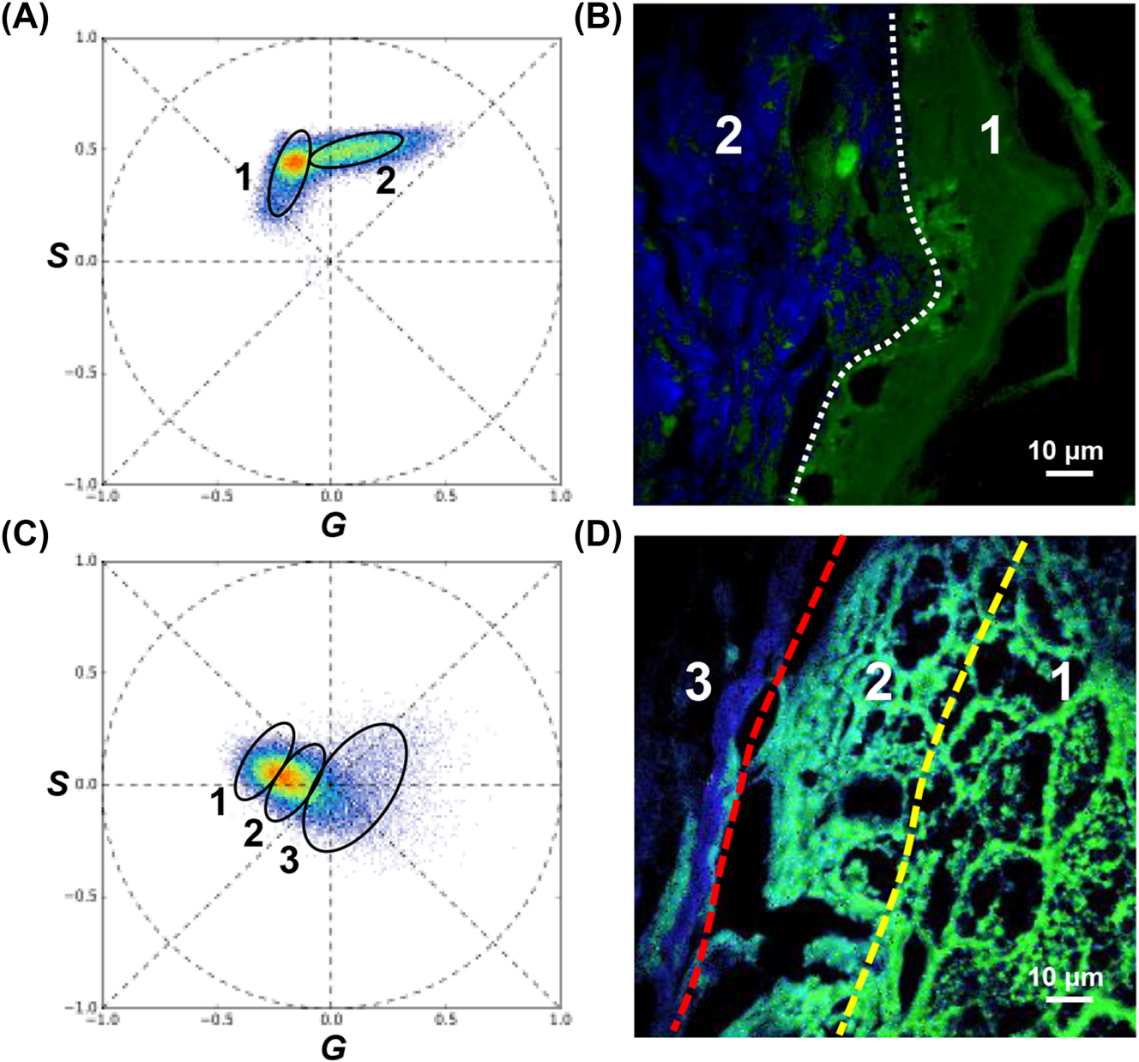
Images of normal tissue and BCC analyzed by spectral phasor. (A) Spectral phasor plot for normal tissue; (B) RGB image of spectral segmentation of normal tissue; (C) spectral phasor plot for BCC; (D) RGB image of spectral segmentation of BCC.

To study the fluorescence spectra and morphological features of skin tissues, we employed a spectral phasor analysis that allowed for the complete segmentation and visualization of structural units with similar spectral features [20], [22], [39]. In the spectral phasor plot for the normal tissue (Figure 5(A)), we identified two regions based on the significant spectral differences between the epidermis and dermis. This segmentation enabled us to separate and reconstruct images with different structural signals, which were subsequently color coded and superimposed as shown in Figure 5(B). The resulting images vividly depict the specific structures of the epidermis and dermis and accurately distinguish their boundaries. Furthermore, the spectral phasor plot was also effective in segmenting structures with subtle differences. In the BCC spectral phasor plot (Figure 5(C)), we delineated three spectral regions within the same cluster, assigning green, cyan, and blue colors to the IR, NEIR, and NOIR, respectively. Because the SHG spectrum differed significantly from the TPEF spectrum at 460 nm, the two spectra were further separated in the spectral phasor plot (region 2 in Figure 5(A) and region 3 in Figure 5(C)), indicating that the SHG signals can be considered almost independent of each other. Note that it is nearly impossible for other methods to rapidly separate regions 1 and 2 (Figure 5(D)) owing to their subtle spectral differences. These results demonstrate that the spectral phasor analysis represents a rapid and efficient approach for distinguishing different structures with overlapping spectra.

**Figure 6:**
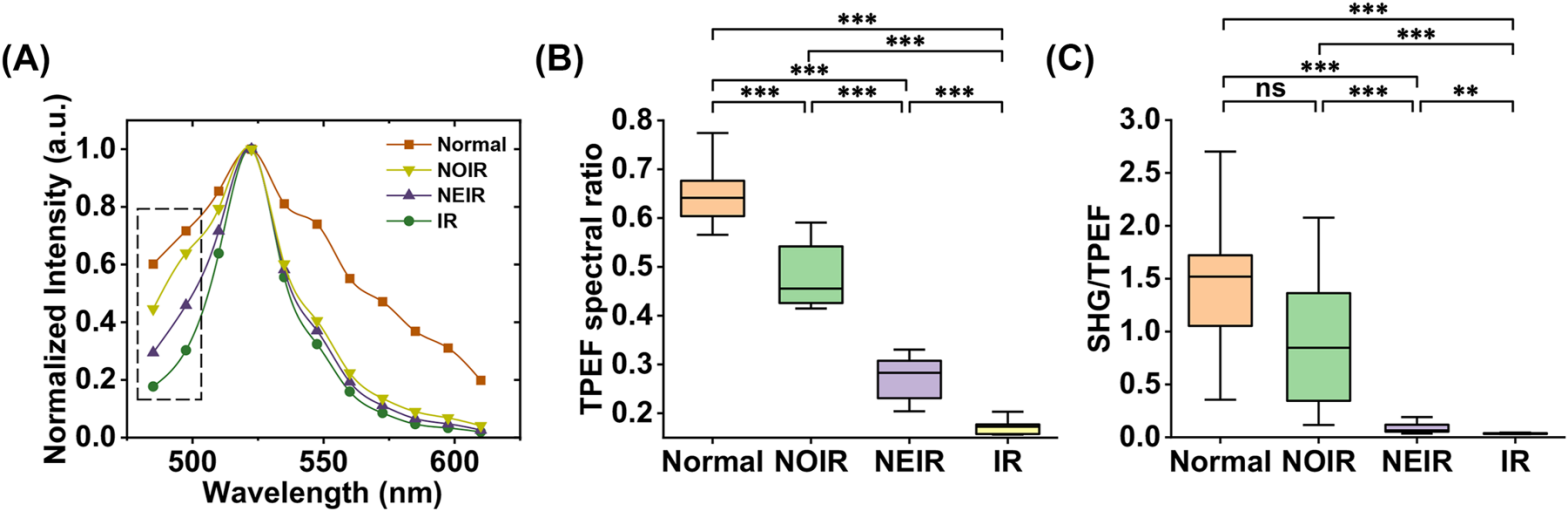
Spectral characteristics of normal and BCC tissues. (A) Comparison of TPEF spectra; (B) ratios of short wavelength TPEF (485 nm ± 6.25 nm) to long wavelength TPEF (522.5 nm ± 6.25 nm) signals; (C) ratios of SHG to TPEF signals. In this image, the error bars denote the SEM, and the notation **** indicates *p* < 0.00001, *** indicates 0.00001 < *p* < 0.0001, ** indicates 0.0001 < *p* < 0.01, * indicates 0.01 < *p* < 0.05, and ns indicates no significant difference via a one-way ANOVA and Tukey’s multiple comparison test.

To investigate the fluorescence spectrum characteristics of skin tissues and quantify their spectral properties, we employed the TPEF signal spectrum to quantitatively identify fluorophores, the TPEF spectral ratio to characterize the spectral differences among different structures, and the SHG/TPEF ratio to quantitatively characterize the degree of collagen decomposition. The spectra of normal and BCC tissues exhibit notable differences in Figure 6(A). In diseased tissues, the fluorescence spectra clearly display an increasing intensity pattern from 485 to 510 nm (dashed box in Figure 6(A)) in the IR, NEIR, and NOIR. This variation is attributed to the differential degrees of the Warburg effect in these regions. The metabolic state and cellular respiratory processes differ significantly between normal and tumor tissues; tumor tissues prioritize meeting metabolic nutrient demands for rapid proliferation rather than efficient ATP production from glucose [36], [40]. This leads to an imbalance in the nicotinamide adenine dinucleotide (NADH) ratio in diseased tissues, resulting in changes in the spectra of different structures and optical redox ratios.

The TPEF spectral ratio was defined to quantitatively assess the differences in the spectra of the various structures at 485 nm (Figure 6(B)). The results indicate a significant decrease in the TPEF spectral ratio from normal tissue to the NOIR, NEIR, and finally IR. This gradual decrease indicates that the degree of aerobic glycolysis during cellular respiration increases and the metabolism of different tumor regions varies as the tumor invades. The SHG/TPEF analysis shown in Figure 6(C) reveals no significant differences among the normal tissue and NOIR. However, the SHG/TPEF ratio differs significantly among the NOIR, NEIR, and IR, suggesting that the breakdown of collagen fibers is progressively enhanced from the outside of the tumor to the tumor border, then to the tumor interior. Collagen fibers within the tumor appear to have undergone near-complete degradation. The differences among the TPEF spectral data indicate that the multidimensional quantitative characterization of endogenous FAD fluorophores and collagen fibers using the proposed system can be applied to study specific processes related to tumor invasion and associated metabolic processes, demonstrating the potential of this technology for clinicopathological analysis.

In summary, these results suggest that fluorescence imaging can be used to observe the breakdown of collagen fibers and study tumor invasion pathways. We observed a clear correlation between the fluorescence lifetime characteristics and the tumor metabolic status. Furthermore, fluorescence spectra can provide complementary information regarding the fluorescence lifetime to facilitate the accurate determination of tumor boundaries. Despite these advantages, FLIM imaging with a 16-Channel PMT still faces challenges, particularly in terms of imaging speed. The determination of accurate fluorescence lifetime values necessitates photon count accumulation, resulting in slower imaging. Notably, the potential of this non-invasive, label-free imaging technique extends to the field of *in vivo* endoscopy [41], where it can be integrated with advances in portable multiphoton endoscopes. Finally, the application of the proposed approach with artificial intelligence algorithms can significantly enhance the utility of spectral- and time-resolved microscopy systems in the biomedical field.

## 4 Conclusions

This study analyzed the morphological changes and microenvironmental metabolic status of BCC by performing a label-free multidimensional quantitative characterization of human skin tissues. Using a series of micro-optical imaging techniques, the different cellular structures of skin tissues such as melanocytes, keratinocytes, and collagen fibers were distinctly identified. The obtained fluorescence lifetime and fluorescence spectra data mutually validated each other, enabling the quantitative analysis of the changes in tumor microenvironment. The results illustrate discernible variations between the average fluorescence lifetime and spectral characteristics at the boundaries and interiors of BCC tumors. We further demonstrated that stronger aerobic metabolism exists at the tumor boundary than in its interior. Thus, the combined analysis of SHG signal intensity, average fluorescence lifetime, and spectral data enabled the accurate determination of the tumor boundary, confirming that the proposed two-photon imaging technique can provide a wealth of pathological information and shows considerable potential for use in cancer diagnosis, tumor boundary assessment, and intraoperative imaging.

## Supplementary Material

Supplementary Material Details
