# Supplementary material for: Multidimensional quantitative characterization of basal cell carcinoma by spectral- and time-resolved two-photon microscopy
Source: Nanophotonics. 2024 Jan 12;13(2):217–27. doi: 10.1515/nanoph-2023-0722 (PMC11501964; doi:10.1515/nanoph-2023-0722)
Supplement: Supplementary file 1 — Supplementary Material Details [file j_nanoph-2023-0722_suppl_001.docx]

**Supplementary materials**

Fig. S1. Fluorescence lifetime and spectra characterization of standard samples. (A) Fluorescence lifetime image of 20 $\mu M$ fluorescein sodium solution. (B) Emission spectra corresponding to (A). (C) Fluorescence lifetime image of urea crystal. (D) Emission spectra corresponding to (C).

Fig. S2. Fluorescence lifetime and spectra characterization of live-cell labeled with different fluorophores. (A) Fluorescence lifetime image of ID8 cells labeled with MitoTracker-Red. (B) Emission spectra corresponding to (A). (C) Spectral phasor plot of (A). (D) Fluorescence lifetime image of ID8 cells labeled with LysoTracker-Green. (E) Emission spectra corresponding to (D). (F) Spectral phasor plot of (D). MitoTracker-Red: 1 μM, 30 min, 37 °C, one wash; LysoTracker-Green: 300 nM, 30 min, 37 °C, one wash.

Fig. S3. Multidimensional optical characterization of BCC. (A) Histological image of H&E staining; (B) Merge image of TPEF and SHG; (C) Comparison of TPEF spectra; (D) Spectrum-coded image of TPEF signals; (E) Spectrum-coded image of SHG signals; (F) Lifetime-coded image.
